# A systematic tale of two differing reviews: evaluating the evidence on public and private sector quality of primary care in low and middle income countries

**DOI:** 10.1186/s12992-017-0246-4

**Published:** 2017-04-12

**Authors:** Jorge Coarasa, Jishnu Das, Elizabeth Gummerson, Asaf Bitton

**Affiliations:** 1 World Bank, KK Birla Marg, Lodhi Estate, New Delhi, India 110003; 2 World Bank, MSN MC3-311, 1818 H Street, NW, Washington, DC 20433 USA; 3 Center for Policy Research, New Delhi, India; 4 The International Center for AIDS Care and Treatment Programs, Columbia University, Mailman School of Public Health, 722 W. 168th Street, 13th floor MSPH, Box 18, New York, NY 10032 USA; 5 Ariadne Labs, Harvard T.H. Chan School of Public Health and Brigham & Women’s Hospital, 401 Park Drive, Third Floor East, Boston, MA 02215 USA

**Keywords:** Systematic Reviews, Quality of Care, Public versus Private care, Primary Health Care, LMIC, Primary Care

## Abstract

Systematic reviews are powerful tools for summarizing vast amounts of data in controversial areas; but their utility is limited by methodological choices and assumptions. Two systematic reviews of literature on the quality of private sector primary care in low and middle income countries (LMIC), published in the same journal within a year, reached conflicting conclusions. The difference in findings reflects different review methodologies, but more importantly, a weak underlying body of literature. A detailed examination of the literature cited in both reviews shows that only one of the underlying studies met the gold standard for methodological robustness. Given the current policy momentum on universal health coverage and primary health care reform across the globe, there is an urgent need for high quality empirical evidence on the quality of private versus public sector primary health care in LMIC.

## Background

Systematic reviews are intended to evaluate a comprehensive body of evidence in a way that minimizes the effect of bias. Moreover, they are often used by policy makers as a shorthand guide to the state of the evidence on a subject, and therefore can have considerable impact. We examined the wide discrepancies in methodologies and findings among two recent systematic reviews that examined quality of ambulatory primary care in low and middle-income countries (LMIC).

Universal health coverage (UHC), defined as the ability to ensure access to health services of sufficient quality while protecting people from financial hardship, has become one of the most important discussions in global health policy today. In 2012, following a UN resolution calling for UHC, researchers, advocates, policy makers and governments have been actively engaged in discussing the meaning, scope and implementation of UHC [1–4]. While few dispute the goal of universal access to quality health care at affordable prices, the best means of achieving this goal remains a subject of considerable debate in all countries, but even more so in low and middle income contexts [5].

One contentious issue within UHC expansion is the role of the private sector in expanding access to health care [6]. Proponents of private ambulatory primary care provision argue that the private sector is already a significant source of health care and can be instrumental in expanding efficient and high quality care to underserved populations [7, 8]. Skeptics claim that the private sector privileges profit incentives over public health needs and is unlikely to provide low-cost, high-quality health care to poor populations [9]. Consequently, expanding and strengthening ambulatory primary care public services should be the focus of efforts towards UHC [7, 10]. Disputants on both sides of the debate claim that the quality of medical care provided by the other sector is inferior [10, 11].

Predictably, policy makers want to see the evidence base for these claims. We have been very interested to see supporters of both perspectives recently referencing two opposing systematic reviews of the literature proving that the private sector does/does not provide better quality care [12–15]. Even more remarkably, the two reviews currently being cited were both published in the same journal, *PLoS Medicine*, roughly within a year of each other. In reviewing the quality of private medical care in LMICs, Berendes, et al conclude *“quality in both provider groups seems poor, with the private sector performing better in drug availability and aspects of delivery of care, including responsiveness and effort, and possibly being more client oriented”* [16]. One year later Basu et al. [17] reach a somewhat different conclusion, stating that *“Studies evaluated in this review do not support the claim that the private sector is usually more efficient, accountable or medically effective than the public sector”*.

Given that both of these reviews were drawing from essentially the same body of literature, it is worrisome that the findings were so different.

To determine how these two groups of reviewers came to contradictory conclusions for a critical policy-relevant issue, we first examined the different methodologies they used in conducting the reviews and found several consequential differences in how study data were collected, aggregated and evaluated in each review. We then analyzed the evidence base itself, pulling the studies cited in both reviews and evaluating them along four methodological standards for robust findings. We conclude that the differing viewpoints of the authors, in part, reflect the fundamental lack of robust evidence on the quality of care in the public and private sector in LMIC. Several conclusions can be supported by existing evidence depending on the weight given to different methodologies, samples or measures of quality. Given the scale and importance of the topic, the research community needs a concerted effort to agree on a defined methodology and gather robust evidence on quality of care in LMIC consistent with the methodology.

### Systematic reviews are not always done systematically

One of the key ways in which a systematic review differs from a literature synthesis is that it follows an explicit and replicable method for selecting and assessing studies. Applying consistent criteria to studies for both inclusion in the review and analysis of findings enables the readers to critically evaluate the reviewer’s conclusions. To that end, we compared the methodology of the two reviews along domains including search strategy, selection criteria, geographic range, methods of study aggregation, representativeness and chosen quality criteria.

Table 1 shows that there were several areas where the reports converged: The search was conducted in December 2010 for both studies for the same geographic range and (with some exceptions) on the same databases. Their exclusion criteria were similar, and importantly, studies with a high risk of bias were excluded in both reviews. We also completed the detailed AMSTAR methodology checklist for Systematic Reviews and graded both studies seven out of a potential score of 11 [18]. Table 1 details each of the checklist items and the scores for the two studies.

**Table 1:** Comparison of review methodologies

Methodological criteria	Berendes et al.	Basu et al.
Search strategy	Keyword search resulting in **8145** abstracts/studies from the following sources: • Medline (Pubmed) • Embase • LILACS • Web of Science, Social Sciences Citation Index and Science Citation Index • Pychinfo • Cochrane Central Register of Controlled Trials (CENTRAL) • Cochrane Methods studies • Cochrane economic evaluations • Cochrane reviews • Other reviews (in Cochrane library) • CSA – Assia, Sociological Abstracts • Econlit	Keyword search resulting in **1178** abstracts/studies from the following sources: • Medline (Pubmed) • Embase • LILACS • Web of Knowledge • African Index Medicus • Eastern Mediterranean Literature – WHO • IndMED • Index Medicus for South-East Asia Region • WHO library database • World Bank documents and reports • UN Children’s Fund • UNDP • Gates Foundation • GFATM • Oxfam International • Kaiser Family Foundation
Inclusion criteria	• Field based studies in LMICs • Directly compare private and public ambulatory care in the same country • In English, German, or French • Scientifically sound data collection methodology for quantitative and qualitative studies	• Study included population data from at least 1 LMIC • Study relevant to review objective • In English, French, Italian, Spanish, Portuguese, or Russian • Scientifically sound data collection methodology for quantitative and qualitative studies
Exclusion criteria	• Unclear or poor sampling criteria • Inadequate sample size/response rate • Risk of bias (through purposive sampling or other study design problems) • Significant errors or omissions in data presentation • Focus on informal providers	• Unclear or poor sampling criteria • Inadequate sample size • Risk of bias via poor design conduct or analysis • Significant errors or omissions in data presentation
Geographic range	LMICs Worldwide	LMICs Worldwide
Aggregation method	Adapted Donabedin (21) classification of quality of care into seven sub-categories of quality of care. Assigned **80 included studies** a numeric score for public and private sector quality of care in each of the sub categories. Aggregated scores into medians and interquartile ranges for public and private providers in each category. Compared median scores between public and private providers.	**Classified 102 included studies** into six categories from WHO framework for health systems assessment. (22) Authors synthesized overall findings in each category.
Date searches conducted	The search was performed in December 2010 and included articles from January 1980 through August 2011	The search was performed in December 2010 and included articles from 1969 – October 2010
*AMSTAR Methodology Checklist (18)*		
Total AMSTAR Methodology Checklist Score	7/11	7/11
*Was there duplicate study selection and data extraction?*	Yes	Yes
*Was a comprehensive literature search performed?*	Yes	Yes
*Was the status of publication (i.e. grey literature) used as an inclusion criterion?*	No	No
*Was a list of studies (included and excluded) provided?*	Yes	No
*Were the characteristics of the included studies provided?*	Yes	Yes
*Was the scientific quality of the included studies assessed and documented?*	Yes	Yes
*Was the scientific quality of the included studies used appropriately in formulating conclusions?*	Yes	Yes
*Were the methods used to combine the findings of studies appropriate?*	No	No
*Was the likelihood of publication bias assessed?*	No	No
*Was the conflict of interest included?*	No	Yes

The bold text signifies important contrasting characteristics of the two systematic reviews

However, we also found three areas of distinct divergence between the two reviews. First, although there was a degree of overlap, they were largely drawing from different bodies of literature. There were only 16 citations in common although one reviewed 102 studies and the other reviewed 80 studies. In part, this is a result of differing inclusion criteria. While Berendes et al. limited their review to comparative studies of ambulatory care, Basu et al. included a wider breadth of non-comparative studies and grey literature in their review.

**Fig. 1:**
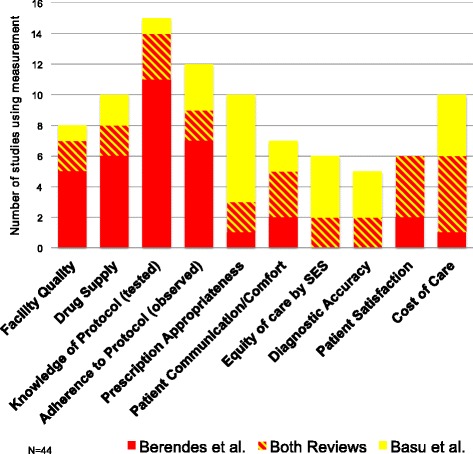
Quality measurements

**Fig. 2:**
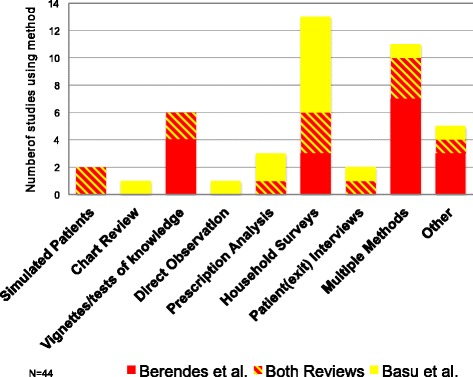
Methods used

Second, the studies included in both reviews defined and measured quality of care in a wide variety of ways. The studies cited in Berendes et al. relied more heavily on measuring medical practitioners’ knowledge of, and adherence to, clinical protocol. The studies included in Basu et al. were weighted more towards measurements of prescription practice and equity of care (Fig. 1). Moreover, the studies cited in Basu et al. were based more heavily on household surveys while Berendes et al. cited more multiple method and facility‐based studies (Fig. 2). Of the eleven multiple methods studies, nine were entirely facility based and two had a facility based component and a household survey component.

Although both reviews covered multiple approaches to measuring and defining quality, the differing frameworks that they adopted resulted in reviews emphasizing different aspects of quality of care, each with multiple potential biases and shortcomings compared to an accepted gold-standard measure of clinical practice using standardized patients. Provider knowledge may be insufficient as a measure of clinical practice as there are significant differences between knowledge and practice, known as the “know-do” gap [19]. For example, among primary providers in India, 21% prescribed potentially harmful treatment for childhood diarrhea when their medical knowledge was tested, but 72% offered these harmful treatments in clinical settings to a standardized patient [20]. Measures of clinical protocol based on direct observations are subject to Hawthorne effects whereby providers may change their behavior when they know they are being observed [21]; they may also be confounded by differences in case or patient-­‐mix across providers. Finally, household surveys rely on accurate recall of specific clinical practices and although recall biases in these household surveys has not been evaluated (to our knowledge), research in other settings shows little correlation between providers’ actions in clinics and what patients themselves say in exit surveys as well a large recall biases in household reports of doctor visits and health expenditures [22, 23].

Finally, the two studies used very different methods of aggregating results. Berendes et al. applied a mathematical scoring method to each study and compared scores between public and private providers across seven categories; Basu et al. categorized findings into six thematic areas and the authors then synthesized and summarized findings under each thematic area. These two very different approaches to synthesis of studies unsurprisingly resulted in divergent conclusions.

Again, it is difficult to claim methodological superiority for one method over the other. Berendes et al. require that the weight given to the (very different) quality measures is identical, so, for example, a reduction of antibiotic use by 10 percentage points has the same implications for patient’s wellbeing as an improvement in the rate of correct diagnosis by the same amount on the same baseline values. Basu et al. allow for greater variation in welfare weights, but at the cost of introducing considerable subjectivity in their synthesis; different readers may reasonably reach different conclusions based on the same underlying evidence, especially when the evidence itself (as we show next) is weak.

### The underlying data are very limited and heterogeneous

The discrepant conclusions by these two reviews also suggest that the underlying evidence itself may be subject to multiple interpretations.

The second part of our examination therefore evaluated the underlying data upon which the reviews were based. We extracted from the complete citation lists of both articles the studies that were written in the year 2000 or later and compared public and private providers directly. Across the two reviews, there were only 44 directly comparative studies in *all* LMIC. While the reviews did not cite every study they examined, the small number of studies cited indicates a limited pool of empirical evidence (as acknowledged by both sets of reviewers in their discussion). In addition, sample sizes in facility-­‐based surveys were small; 70% of the cited surveys reported sample sizes under 250. Sample sizes in the household surveys were larger but these analyses, based solely on patient recall, convey only limited information about the details of clinical consultations.

To assess how many cited studies met a gold standard of robust evidence for quality comparison across the two sectors, we suggested and used four criteria to define a methodologically accurate comparison study (Table 2).

**Table 2:** Minimum methodological criteria for comparing quality of care between public and private sector providers

Criterion	Rationale
*Does the study have a viable comparison group?*	Documenting good or poor quality of care without knowing how good the care is amongst equivalent providers in the community isn’t that informative.
*Does the study control for patient mix?*	Different types of providers are frequented by different types of patients. Alternately, the same patient may select provider sector based on what s/he perceives to be the problem. This can bias comparisons because the compared providers aren’t treating patients with the same type or severity of problems.
*Does the study control for differences in provider mix?*	Public sector providers generally have a narrow range of certified medical qualifications. Private sector includes people with those same qualifications plus pharmacists, traditional healers, informal providers with no medical training, and public providers moonlighting in private sector. Comparing providers without taking into account differences in qualifications confounds the effect of the sector with the effect of medical training.
*Does the study control for differences in resources?*	Different types of providers have different resources available to them – both financially and in terms of equipment and trainings, etc. Cost analyses should be included in the analysis of quality of care and should consider subsidies that providers receive as well as charges and costs to patients.

1. There should be a viable comparison group.
2. The analysis should control for patient and case-­‐mix either through direct elicitation of patient characteristics (for instance, by using an exit survey of patient characteristics matched to clinical interactions) or, preferably, through the use of standardized patients.
3. The analysis should control for provider mix as the population of providers can differ across the public and private sector and comparisons based on provider populations may confound differences in care by sector with differences in care by medical training and;
4. The authors should consider differences in provider resources as the resources available to provide care may differ across sectors.

The application of each criterion reduced the number of studies significantly. Of 44 studies to begin with, 23 made any attempt to control for patient mix. Of these, 12 attempted to control for provider mix and ultimately, we were able to find only one study that met all the minimum criteria. This study, by Pongsupap and Van Lerberghe [24], used standardized patients to evaluate responsiveness, patient centeredness and clinical accuracy among outpatient care providers in Bangkok. The use of standardized patients represents a gold standard for research on patient-provider interactions in LMIC for a number of reasons. First, data from chart abstractions are mostly absent in LMIC. Second, standardized patients allow researchers to evaluate the treatment choice against a medically objective notion of ideal case management, as the researchers know *a priori* the illness that the standardized patients presented with. Third, data from standardized patients are free from most confounders, and also reduce known Hawthorne effects. Pongsupap and Van Lergerghe [24] took care to assess “similar” providers in the public and private sector and provided data on available resources in both sectors. Both reviews cite this study.

However, even this study can be faulted. First, “similar” qualifications may not necessarily imply the “same” providers as those who choose to practice in the public sector may have different levels of knowledge and motivation relative to those in the private sector (an alternate research design would look at the same provider in both public and private sector practices). Second, the study relies on only one simulated set of symptoms, which *none* of the providers diagnosed accurately, so that the only variation was the use of “unnecessary” medications, which was the same across both sectors. The case was “too hard” to assess therapeutic differences between the two sectors.

With more strict criteria for methodological robustness, the entire evidence base for comparative quality in public versus private provision of care reduces to a single study for a single tracer-condition in Bangkok, Thailand, where there was no variation in diagnostic accuracy but the private sector was more patient centered in their approach.

## Discussion

Health care policy researchers know far less about the quality of publicly versus privately provided primary care than it would seem at first glance with two recent systemic reviews. Unfortunately, it is often too easy for policy makers to interpret the findings of a systematic review as the last word on a subject. As we have demonstrated, different review methodologies can lead to drastically different conclusions when the actual body of evidence is so limited.

Systematic reviews are gaining prominence in health policy and clinical medicine; over 2500 are produced annually [25], and some commentators refer to them as a gold standard of evidence summary [26]. Notably, though, the quality of many of these systematic reviews is poor, and large heterogeneity exists in their search strategies and use of exclusion criteria [25].

Duplicate systematic reviews and meta-analyses are also on the rise. A 2013 study found at least 2/3 of these reviews had a duplicate or overlapping companion study [27, 28] Whether these duplicate reviews add a layer of validation and uncover new data, or obscure clarity on the state of the evidence is hotly debated. Petticrew [29] has argued that systematic reviews of complex interventions or policy questions might be better served with more narrow questions, stronger inclusion criteria, and a Bayesian approach to knowledge inference [29].

Our belief is that the problem lies less with the reviews themselves than with the quality of the underlying data. We found strong heterogeneity within the reviewed studies of outcome, intervention, and context. If there were a large body of good evidence with consistent interventions, slight differences in review methodology might not have such consequences. As it is, we find that there is a very small pool of highly robust empirical studies on public versus private quality of ambulatory primary care in LMIC. There is an urgent need for well-designed comparative work that is rooted in direct observation of patient-provider interactions or standardized patients, controls for patient mix and provider mix, and evaluates cost and differential resources. Without better data, policy makers working towards universal health coverage will need to make critical decisions based upon evidence that is both too weak and too scarce.

Systematic reviews were originally designed for understanding narrow clinical topics that had a robust research base of information consisting of relatively homogenous clinical trial designs. Increasingly they are being used to summarize disparate evidence regarding complex, multi-dimensional organizational changes. The primary care research community needs to reach some consensus on how best to use, adjust, and interpret these types of reviews in order to inform policy makers and implementers on critical policy decisions.

## Conclusion

Systematic reviews are widely viewed as an accurate synthesis of multiple studies that provide unambiguous guidance for policy. However, multiple systematic reviews on the same topic often lead to contradictory conclusions, as is the case for the quality of public and private care in low and middle-income countries. We examined two such reviews that appeared in the same journal around the same time, each leading to a different conclusion. Returning to the original studies, we find that weaknesses in the underlying evidence, rather than the rigor of the reviews themselves led to reasonable disagreements. In cases where questions are complex, outcomes are multidimensional and results are sensitive to study methodologies, a better understanding of what constitutes a ‘high quality study’ and therefore should be highly weighted in the review is critical. In such cases, systematic reviews could present a wider description of the evidence, at least until there is a wider consensus on what is to be measured and how.

## Abbreviations

AMSTAR: A measurement tool to assess systematic reviews
LMIC: Low and middle income countries
UHC: Universal health care
